# In search of the best analysis regarding treatment for meningoencephalitis of unknown origin in dogs

**DOI:** 10.3389/fvets.2022.1062114

**Published:** 2023-01-04

**Authors:** Mark Lowrie

**Affiliations:** Dovecote Veterinary Hospital, Derby, United Kingdom

**Keywords:** meningoencephalitis of unknown origin (MUO), survival, randomized control trial, canine, death, per-protocol analysis, intention-to-treat analysis, immunosuppression

## 1. Introduction

The treatment of meningoencephalitis of unknown origin (MUO) continues to receive an enormous amount of scientific attention every year. In order to provide their patients with the best treatment options, practitioners should be up to date on the latest research findings. Veterinary practitioners are charged with critiquing the medical literature to remain updated on the most recent developments in management. To facilitate a patient's care, the practitioner must bring the best available evidence to the consultation and present it in clear terms to educate the owner and empower them to make the best decision for their pet. The latest studies are delivered in research journals to the veterinary profession having undergone extensive peer review to ensure they convey a high standard, scientifically relevant content. There remains an onus upon the practitioner to scrutinize these journal's offerings to ensure bias is absent and quality is assured. In the search for the best management of MUO, numerous studies have been published with wide ranging survival times and conclusions. The reality of all of these research items is that the quality of the evidence base is poor consisting predominantly of retrospective studies and the occasional prospective, infrequently controlled, analysis (1). This article will review the intention-to-treat (ITT) principle and its converse, per-protocol analysis, to demonstrate how using the wrong method of analysis in MUO treatment trials can lead to a significantly biased assessment of the effectiveness of an intervention.

## 2. Randomized controlled trials

Randomized controlled trials (RCT) are the best way to establish causal relationships between interventions and outcomes (2, 3). It is these trials that are completely lacking in the treatment of MUO. The main purpose of randomization is to prevent selection bias and generate groups that are comparable to one another. When randomization is correctly conducted, it results in groups with balanced prognostic variables (variables which influence the development of the outcome under study). This allows veterinarians to make an accurate, convincing argument that a difference in outcome between two (or more) prognostically balanced groups, excluding the intervention, can be attributed to the intervention.

The gold standard method to establish causal relationships between interventions and outcomes is generally considered to be randomization. However, this method does not guarantee the complete absence of bias. When the data are analyzed incorrectly, bias can occur even when a valid random allocation sequence is implemented (e.g., utilizing a table of random numbers or computer software that generates a random sequence). In order to ensure that randomization remains accurate during the study and analysis, it is imperative to maintain its integrity. When the veterinarian fails to give patients the proper evaluation based on their original assignment, results may be incorrectly interpreted and biased.

When randomization is disrupted, bias can inadvertently alter the analysis and outcome. This means that an investigator's aim should be to maintain prognostic balance between groups at all times during the study, from admission, allocation, intervention, follow-up through to analysis. One method of assessing patients based on their initial assignment is called ITT (4). This article details a standard MUO hypothesis and describes the design and interpretation of the associated study illustrating how it can be misleading to interpret results from RCTs without applying the ITT concept.

### 2.1. Why use intention-to-treat in an RCT?

**Figure 1:**
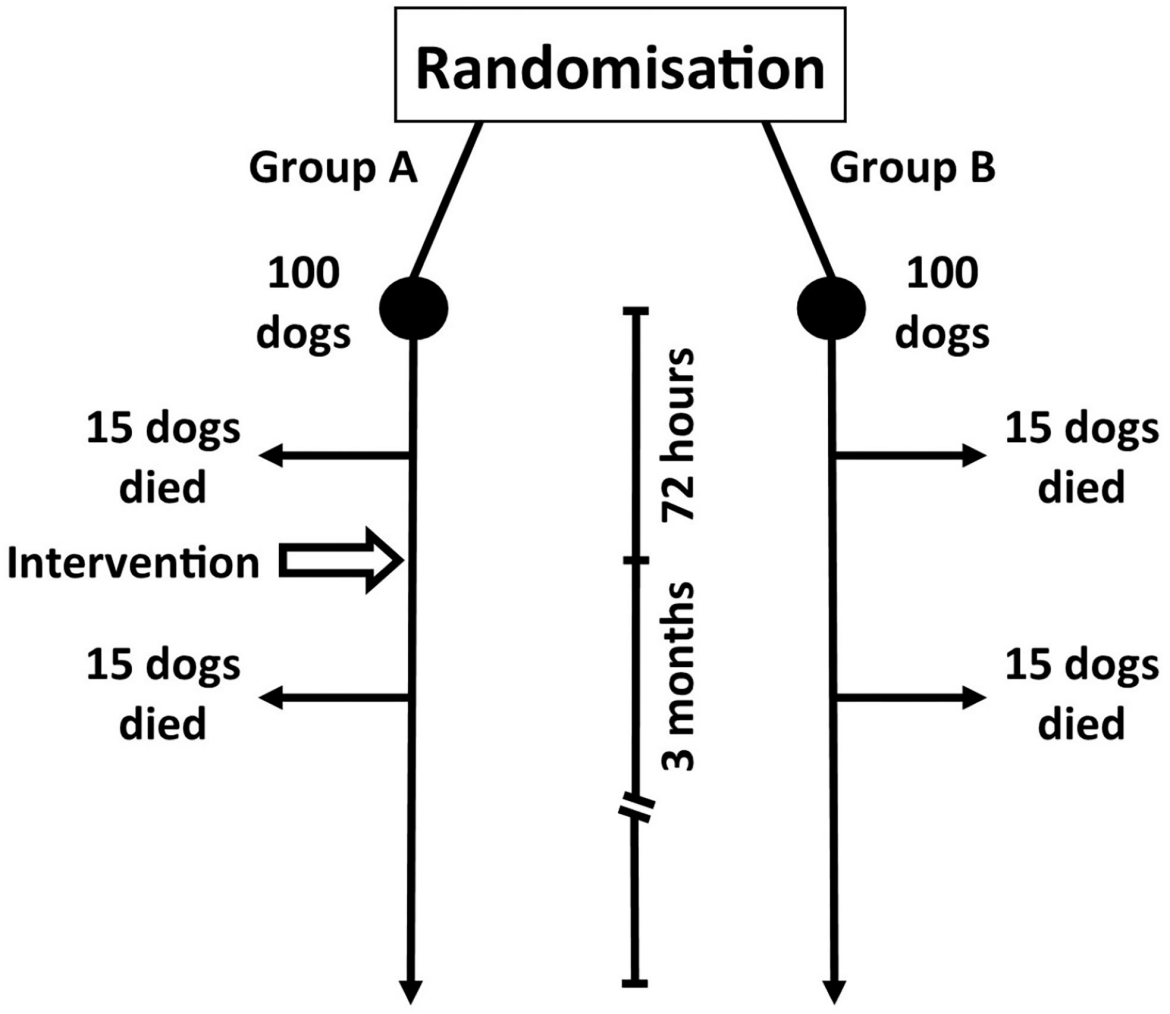
Hypothetical prospective study evaluating the efficacy of an intervention (Group A; medical management plus immunosuppressive adjunct) vs. control (Group B; medical management only) in dogs with meningoencephalitis of unknown origin.

In order to address this question we will consider an example. We want to evaluate the effectiveness of adding adjunctive immunosuppressive medication (intervention) to prednisolone (the conventional treatment) in preventing death (outcome) in patients with MUO (Figure 1). In our example we manage to recruit 200 dogs to our RCT and these patients are split equally into two groups; group A will receive the intervention (prednisolone + adjunct) and group B is our active control (prednisolone only). We will evaluate outcome after 3 months.

It is important to realize that the nature of our study design is that we have a short period of 72 h between randomization (i.e. point of diagnosis) and intervention. At the 3-month follow-up, 30 dogs in group A have died (the study's primary outcome measure). Fifteen of these dogs died within 72 h of enrolment whereas the 15 other dogs died 72 h−3 months later. A similar outcome is seen with group B (Figure 1).

Now consider that adding this immunosuppressive medication (intervention) does not prevent our primary outcome (death). We can consider this as the “truth.” Our work should therefore be to perform randomized trials to determine this “truth.” The way we choose to analyse the data will determine if we can arrive at an unbiased conclusion (finding the truth). Using the right analysis, we should determine that the adjunctive immunosuppression is unsuccessful, whereas in the wrong analysis, we will come to a false and biased assumption that additional immunosuppression improves mortality.

### 2.2. Analysis using a per-protocol method

The first step in our analysis is to consider which dog received the protocol-allotted intervention. This is a method known as per-protocol analysis (5). This may seem an odd thing to do as all dogs in the intervention group could be assumed to have received the intervention. The reality, however, is that only 85 dogs in group A received the intervention; 15 dogs died before it could be administered. Based on this method of analysis, the mortality rate is 0.18 or 18% (15/85) whereas the death risk for our control group is 0.3 or 30% (30/100).

The relative risk of death refers to the ratio of the intervention group's death risk to the control group's death risk. In this case, the risk ratio is 0.59 (0.18 divided by 0.3). To determine the relative risk reduction of death we must subtract this value from 1 to give 0.41 or 41%. In other words, data analysis using a per-protocol method may mean that a veterinarian would conclude that the adjunctive immunosuppression reduces a dog's death risk by 41%.

We know, however, that the adjunct has no effect on reducing mortality. Analyzing the intervention in this manner would be to grossly misinterpret and inaccurately misrepresent the results. Clinical practice based on such an inaccurate interpretation would result in patients suffering from receiving an additional immunosuppressive medication no benefit.

There is another method of analyzing this data that considers the dogs based on the treatment they had administered rather than the group they were originally designated. This method, called as-treated analysis (3), also suffers from the introduction of selection bias and thus removes any advantage gained by randomization.

Using this strategy, dogs receiving the adjunct within the control sample (whatever the reason), are considered to be participants of the intervention arm, and those allocated within the adjunctive treatment group that do not receive the adjunct are considered to be participants of the control arm. When assessing an RCT's findings, the use of as-treated and per-protocol methods increases the possibility of bias. To avoid this trap, there is a strategy to analyse data from an RCT that will not result in this kind of erroneous conclusion. Intention-to-treat (ITT) analysis is the name of this technique.

### 2.3. What is intention-to-treat analysis?

Regardless of all the mishaps that can happen during a study, ITT means that one should analyse all dogs allocated randomly to one of the treatments collectively as representing that treatment, whether they received only some or even none of the treatment. In doing this it ensures that randomization remains intact and confounding variables are reduced preserving the prognostic balance. Therefore, using the ITT principle in our study requires uniformly complete follow-up of all dogs (as far as possible) and assigns credit for all positive or negative events and outcomes to the randomized treatment group, regardless of the changes in treatment actually received by those dogs.

Looking back at our study, a dog in the intervention group faces a 30% chance of dying. This calculation includes the 15 dogs that died (the study's primary outcome) before the adjunctive medication was administered. Control dogs have a 30% (0.3 or 30/100) mortality. Compared to the control group, dogs receiving the intervention have a relative risk of death of 1 (0.3/0.3) with the relative risk reduction being zero.

Accordingly, the ITT principle is correct to conclude that the immunosuppressive adjunct is ineffective. It may be contested that it is unreasonable to include the 15 dogs that died before being administered the immunosuppressive agent. However, this is an entirely reasonable practice. It disturbs the prognostic balance achieved by randomization to remove dogs from one arm or the other. A RCT that excludes dogs is more likely to be biased (6).

Studies investigating treatment protocols in MUO often fail at this first hurdle. It is well-recognized that MUO carries a high risk of death within the first 72 h (7–9). Patients randomized after this time frame will have a better prognosis. The period between randomization and group treatment allocation should also always be included in analysis. It is all too frequent that patients with MUO die in these initial hours before treatment is administered and researchers exclude them from analysis; this is a particular problem when retrospective studies gather information on outcome (10, 11). Timing of accession and randomization in MUO studies will directly affect the perceived efficacy of the treatment in question. Those patients lost before randomization will not be prognostically identical to those who remain. The omission of patients that die before the adjunctive treatment is given would cause fewer deaths to be counted and consequently the as-treated analysis would be biased in favor of adjunctive immunosuppression. An example of this is shown in a retrospective study evaluating procarbazine as adjunctive therapy for MUO (12). The median reported survival time in 21 dogs was 14 months according to an as-treated analysis. However, this study is to be commended as it reports that 11 dogs failed to survive to receive treatment giving the study a 34% mortality rate (11/32). Including these deaths in an ITT analysis would significantly decrease the headline median survival time. Failure to acknowledge these deaths sets a dangerous precedent. Studies reporting as-treated analysis for MUO are often conspicuous by their relatively longer median survival times than those published in prospective reports. Once randomization has been performed, dogs (or specifically owners) that fail to adhere to the group's protocol can vary in many regards, not just in the failure to receive the allotted treatment. It is well-known that adherent owners tend to own dogs that have a better outcome than owners of dogs that are non-adherent. This phenomenon is coined the “healthy adherer” effect (13). An ITT analysis maintains the prognostic balance achieved from randomization, thereby minimizing the risk of bias being introduced by unwittingly creating and comparing groups with potentially confounding variables.

The application of ITT allows us to gain an unprejudiced evaluation of the efficacy of a treatment on preventing the study outcome (death). When an intervention decreases the chance of death but adherence to the study protocol is reduced, an ITT analysis underestimates the benefit that medication will give to those dogs that adhere to the treatment schedule. As with a per-protocol or as-treated analysis, ITT yields a more accurate and unbiased efficacy although the magnitude of its effect may be underestimated.

## 3. Conclusion

Large scale randomized clinical trials for MUO face a number of challenges, most notably the variable nature of the condition, which makes defining robust entry criteria and outcomes challenging. Secondly, MUO often results in euthanasia of those affected. However, the timing and nature of this decision can differ given individual circumstances of the owner and attending practitioner that in turn will reduce the validity of death as a robust marker in treatment efficacy. The way ahead is difficult, but all future MUO studies should consider the level of evidence presented before forming concluding remarks.

## Author contributions

The author confirms being the sole contributor of this work and has approved it for publication.
